# Psychosocial Burden of Duchenne Muscular Dystrophy on Caregivers in Australia: A Cross-Sectional Study

**DOI:** 10.3390/healthcare14193352

**Published:** 2026-10-08

**Authors:** Hansoo Kim, Arun Jones, Melinda Fulthorp, Daniel Tan, Leonard Lee

**Affiliations:** 1 School of Medicine and Dentistry, Griffith University, Gold Coast, QLD 4222, Australia; 2 Faculty of Health Science & Medicine, Bond University, Gold Coast, QLD 4226, Australia; 3 Roche Products Pty Limited, Sydney, NSW 2000, Australia

**Keywords:** Duchenne muscular dystrophy, carer burden, quality of life

## Abstract

Background: Duchenne muscular dystrophy (DMD) is a rare, progressive neuromuscular disorder associated with substantial psychosocial demands for family caregivers. Australian evidence remains limited. This study quantified caregiver burden, health-related quality of life, depressive symptoms and work impairment, with a primary comparison by ventilation status and exploratory analyses across functional stages. Methods: A cross-sectional online survey was conducted from February to March 2025 through national advocacy groups. Validated instruments included the Oberst Caregiving Burden Scale (OCBS), Work Productivity and Activity Impairment Questionnaire (WPAI), EuroQol-5 Dimensions-5 Levels (EQ-5D-5L) and Beck Depression Inventory-II (BDI-II). Responses were screened using IP address, completion status and internal consistency before reanalysis. Results: Seventy-three caregivers were included. Most were mothers (67%) and were aged 35–44 years (51%). Mean OCBS difficulty scores were higher among caregivers of ventilated than unventilated patients (39.6 vs. 33.1, *p* = 0.023). Clinically relevant depressive symptoms (BDI-II ≥ 14) were observed in 13 of 19 caregivers of ventilated patients (68.4%) and 46 of 54 caregivers of unventilated patients (85.2%). The odds ratio was 0.38 (95% CI 0.095–1.59, *p* = 0.17). After covariate adjustment, higher OCBS difficulty and time scores remained associated with lower EQ-5D-5L scores, whereas BDI-II and WPAI domains did not. Global interaction tests did not provide evidence that associations differed by DMD stage. Conclusions: Caregiver psychosocial burden was substantial in both ventilation groups. Ventilation status was associated with caregiving difficulty, but the clinical importance of the observed difference is uncertain. Depressive symptoms were common irrespective of ventilation status. These cross-sectional findings support attention to caregiver wellbeing across DMD care but do not establish change over the disease course.

## 1. Introduction

Duchenne muscular dystrophy (DMD) is a rare X-linked recessive neuromuscular disorder caused by mutations in the dystrophin gene, characterised by progressive muscle degeneration with secondary respiratory and cardiac complications that ultimately determine prognosis [1]. Clinical manifestations typically emerge in early childhood, presenting as delayed motor milestones and progressive weakness. Loss of ambulation generally occurs during adolescence and is followed by progressive cardiorespiratory decline. Currently, no cure exists, and clinical management remains supportive [1,2].

Survival in Australian patients with DMD has improved over the past five decades [2]. Earlier diagnosis and wider use of corticosteroids, cardioprotective therapy and non-invasive ventilation have contributed to this improvement. Median survival increased from approximately 18 years among boys born before 1970 to around 24 years among those born in the 1990s. Earlier Australian data similarly reported a mean age at death of approximately 20 years during the 1960s–1980s [2]. More patients now survive into adulthood, extending the duration and complexity of family caregiving.

The estimated global birth prevalence of DMD is 19.8 per 100,000 live male births, with a point prevalence of 7.1 per 100,000 males [3]. Australian birth prevalence estimates are similar, ranging from 18.6 to 22.7 per 100,000 live male births [1].

DMD also has substantial effects on family caregivers. International studies report adverse effects on physical and mental health, family functioning, sleep, employment and productivity [4,5]. Caregivers commonly report stress, anxiety and depression, and health-related quality of life is often below population norms [4,5].

Caregiver burden is generally greater at more advanced functional stages, when loss of ambulation, respiratory support and medical complications increase care requirements [4,5]. However, these associations are not uniform. Some outcomes, including anxiety, may relate more closely to caregivers’ perceptions of patient wellbeing than to functional stage alone [4,5].

Australian research has focused mainly on prevalence, patient health-related quality of life and direct economic burden, with less attention to caregiver mental health and wellbeing [1]. Longer survival further increases the need to understand the effects of sustained caregiving into adulthood.

Ventilation dependence is a clinically meaningful marker of advanced DMD because it reflects increasing respiratory support requirements and caregiving complexity. It was therefore examined as the principal clinical comparator. This study aimed to quantify the psychosocial burden experienced by caregivers of people with DMD in Australia, with a primary comparison of caregiver outcomes according to ventilation status and exploratory analyses examining associations across functional disease stages.

## 2. Methods

### 2.1. Study Design and Recruitment

This study employed a cross-sectional online survey design. The study was conducted in accordance with the Declaration of Helsinki and was approved by the Griffith University Human Research Ethics Committee (GUHREC, GU Ref No. 2024/709). All participants provided informed electronic consent prior to participation. Caregivers of individuals with Duchenne muscular dystrophy (DMD) were recruited through national muscular dystrophy patient advocacy groups, supplemented by snowball sampling whereby recipients were encouraged to share the survey with other caregivers in their networks. The online survey was administered via Qualtrics Core XM (accessed 2025; Qualtrics, Provo, UT, USA) between 17 February and 4 March 2025. At the conclusion of the survey, participants were given the option to provide their email address to receive compensation at fair market value in recognition of their time and effort. Because invitations were distributed through advocacy groups and participant sharing, the total number of invitations and a response rate could not be determined. DMD diagnosis was reported by caregivers and was not independently verified against clinical or genetic records. More than one caregiver from the same household could participate, and household membership was not independently verified.

Before reanalysis, repeated IP addresses were examined alongside completion status and responses to patient and household items. A shorter incomplete entry that matched a completed response was excluded, as were all entries from repeated IP addresses containing contradictory patient or household information. Pairs that were internally consistent and plausibly represented two caregivers from the same household were retained because individual identity could not be verified. This process excluded 10 records. The audit is summarised in Supplementary Table S14.

No formal target sample size or a priori power calculation was specified because the study was exploratory. Reporting followed the CHERRIES and STROBE checklists provided in the Supplementary Materials.

### 2.2. Survey Content

Demographic and clinical information was collected for caregivers and patients. The following validated instruments assessed caregiver outcomes.

#### 2.2.1. Oberst Caregiving Burden Scale (OCBS)

The OCBS is a 15-item measure of the time required and perceived difficulty of caregiving tasks across physical, emotional and social domains [6,7,8]. Each task was rated separately for time and difficulty on a five-point scale. Time responses ranged from 1 (none) to 5 (a very large amount), and difficulty responses ranged from 1 (no difficulty at all) to 5 (extremely difficult). Each subscale has a summed score of 15–75, with higher scores indicating more time or greater difficulty.

#### 2.2.2. Work Productivity and Activity Impairment (WPAI)

The WPAI is a validated six-item measure of work productivity and activity impairment [9,10]. The questionnaire records employment status, hours missed because of caregiving, hours missed for other reasons, hours worked, presenteeism and impairment in regular activities. Standard scoring produces percentage measures of productivity loss and activity impairment, with higher values indicating greater impairment.

The WPAI assesses impairment during the preceding seven days. Presenteeism and activity impairment are rated from 0 (no effect) to 10 (completely prevented work or regular activities, respectively). Scores are expressed as percentages, with higher values indicating greater impairment. Under standard WPAI scoring, absenteeism, presenteeism and overall work impairment are applicable to respondents in paid employment, whereas activity impairment is applicable regardless of employment status. Presenteeism additionally requires that the respondent worked during the recall period. Hours missed from work for reasons other than the condition of interest (Question 3) do not enter the standard calculations of absenteeism, presenteeism or overall work impairment.

#### 2.2.3. EuroQol-5D-5L (EQ-5D-5L)

The EQ-5D-5L is a standardised measure of health-related quality of life [11]. It assesses mobility, self-care, usual activities, pain or discomfort, and anxiety or depression across five levels of severity. Responses describe health on the day of survey completion and form a five-digit health-state profile.

EQ-5D-5L health states were converted to utility scores using the Australian value set developed by Norman et al. [12].

#### 2.2.4. Beck Depression Inventory (BDI-II)

The BDI-II is a 21-item screening measure of depressive symptom severity during the preceding two weeks [13]. Each item is scored from 0 to 3, producing a total score in which higher values indicate greater symptom severity.

For the present analysis, a BDI-II score of ≥14 was used to identify clinically relevant depressive symptoms. This threshold was selected for screening in a non-psychiatric caregiver population and does not constitute a clinical diagnosis of depression [14].

### 2.3. DMD Staging

Patients were categorised into seven stages of disease progression (0–6) according to caregiver responses. The seven-stage classification was adapted from the DMD natural-history framework described by Broomfield et al. [15]. Staging was based on functional criteria ranging from “none of the listed impairments” (Stage 0) through to “full ventilation support required” (Stage 6). Patients were assigned the stage equal to the highest criterion met by the patient. Refer to Table 1.

### 2.4. Statistical Analysis

Survey responses were summarised using means, standard deviations, medians and proportions, with graphical presentation where appropriate. Differences in continuous outcomes between caregivers of ventilated and unventilated patients were evaluated using Welch’s two-sample *t*-tests. Missing observations were handled using available-case analysis. Regression sample sizes are reported in Supplementary Tables S1–S13. Multivariable linear regression models were fitted in the overall sample to examine associations between each caregiver-reported outcome measure and caregiver quality of life measured using the EQ-5D-5L. Models for OCBS and BDI-II included DMD stage, caregiver age group, caregiver sex, patient age and caregiver employment status. Models for WPAI included caregiver age group, caregiver sex and patient age because work-related outcome samples were smaller and were restricted by employment and hours worked. Relationship to the patient and number of adult carers were considered, but the available sample did not support more detailed models without over-parameterisation. Within-stage regression models remained unadjusted because of the small samples in several stages. For each measure, an expanded model containing an interaction between the caregiver-reported measure and DMD stage was compared with a reduced additive model using an F-test. Given the small and uneven stage-specific samples, these analyses were exploratory. All analyses were performed in R version 4.3.1 (R Foundation for Statistical Computing, Vienna, Austria), with statistical significance defined as *p* < 0.05.

## 3. Results

### 3.1. Demographics

A total of 73 caregivers provided patient information. The mean age of patients was 10.8 years (SD 6.2). Symptoms were first reported between one and four years of age for 53 patients (73%). At survey completion, 54 patients (74%) were not using ventilation support (Table 2).

A total of 73 caregivers were included in the demographic analysis. Most were female (66%), with mothers comprising 67% of respondents and fathers 27%. Just over half were aged 35–44 years (51%), and 75% reported two adults providing care in the household. Fifty-one caregivers (70%) were in paid employment and 22 (30%) were not working (Table 3).

**Table 3:** Sociodemographic and employment characteristics of caregivers of patients with DMD.

Caregiver Characteristics (*n* = 73)	Frequency (%)
Age, years (%)	
18 to 24	2 (2.7%)
25 to 34	12 (16%)
35 to 44	37 (51%)
45 to 55	14 (19%)
55 to 64	8 (11%)
Gender	
Female	48 (66%)
Male	25 (34%)
Relationship to person with DMD	
Father	20 (27%)
Mother	49 (67%)
Sibling	4 (5.5%)
Number of adults giving care in household	
Two adults giving care in your household (you and your partner)	55 (75%)
One adult giving care in your household (you are the only carer)	14 (19%)
Other/I’d prefer not to answer	4 (5.5%)
Employment status	
Working for pay	51 (70%)
Not working	22 (30%)

**Figure 1:**
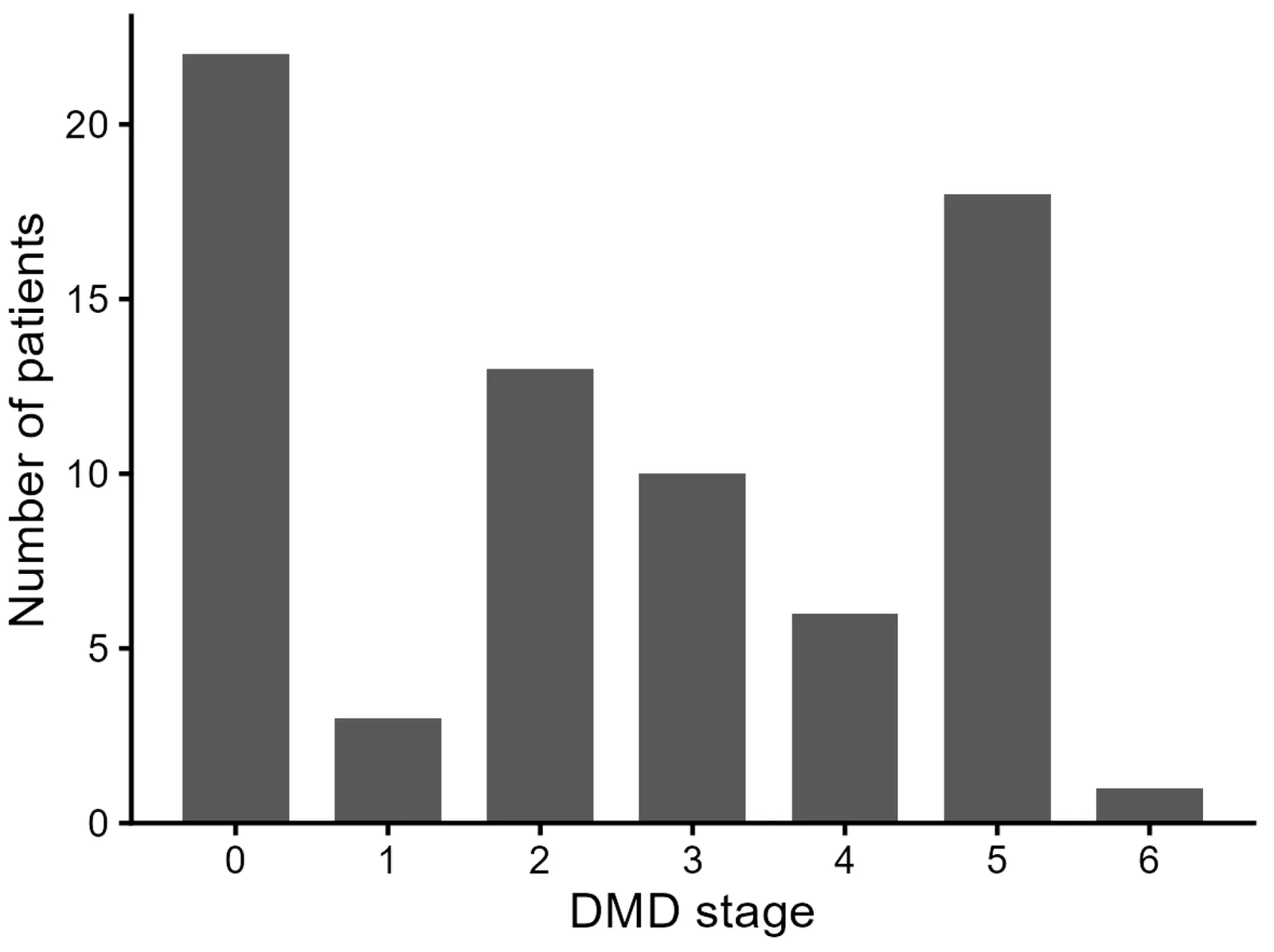
Distribution of patients across DMD functional stages (*n* = 73).

DMD stage distribution was Stage 0, 22 patients (30%); Stage 1, 3 (4%); Stage 2, 13 (18%); Stage 3, 10 (14%); Stage 4, 6 (8%); Stage 5, 18 (25%); and Stage 6, 1 (1%) (Figure 1).

**Figure 2:**
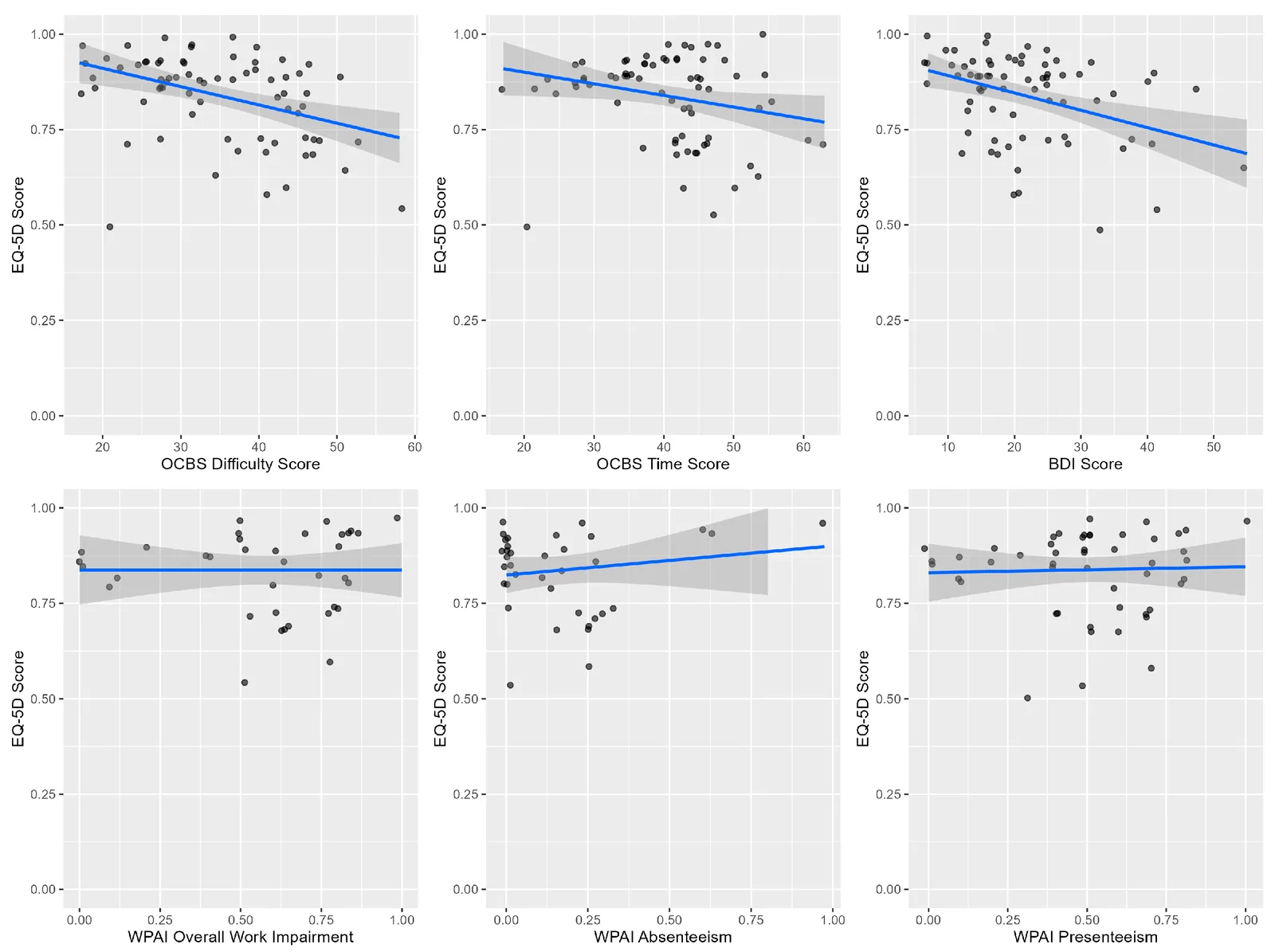
EQ-5D scores plotted against other caregiver-reported outcome measures. Line of best fit: Ordinary Least Squares (OLS) univariate regression.

As shown in Figure 2, higher OCBS difficulty, OCBS time and BDI-II scores were associated with lower EQ-5D-5L scores in unadjusted analyses. Associations between EQ-5D-5L and WPAI outcomes were weak. In adjusted models, OCBS difficulty (beta = −0.0048, 95% CI −0.0075 to −0.0021, *p* < 0.001) and OCBS time (beta = −0.0030, 95% CI −0.0058 to −0.0002, *p* = 0.036) remained associated with lower EQ-5D-5L scores. BDI-II and WPAI domains were not statistically significant after adjustment (Supplementary Tables S8–S13).

**Figure 3:**
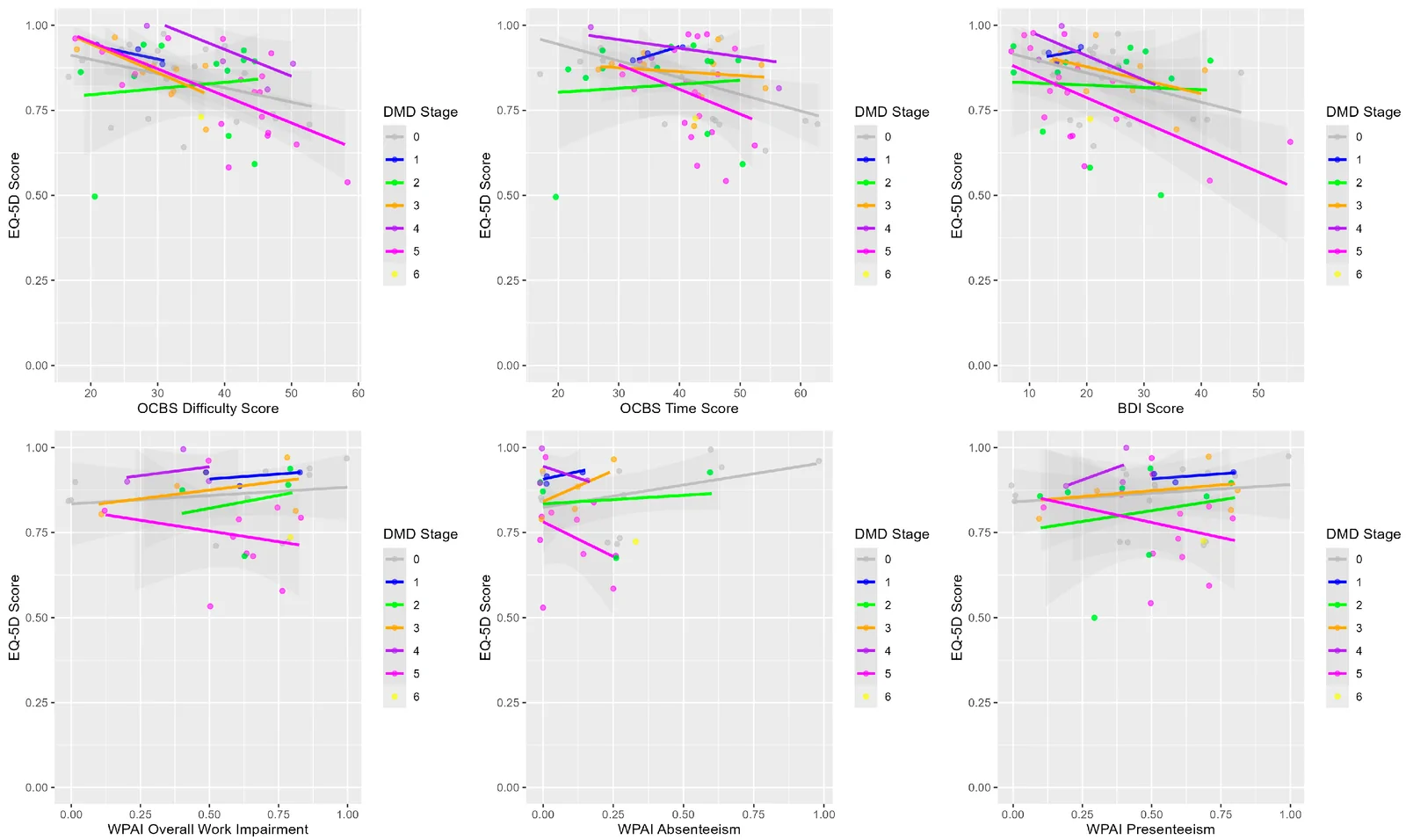
Stage-stratified associations between EQ-5D scores and other caregiver-reported outcome measures. Line of best fit: OLS within-stage univariate regression (stratified). Global interaction tests compared models containing an interaction between DMD stage and each caregiver-reported measure with reduced additive models. None improved model fit, with *p* values ranging from 0.25 to 0.95 (Supplementary Tables S1–S6).

Figure 3 presents exploratory, unadjusted stage-stratified associations between each caregiver-reported outcome measure and EQ-5D-5L. Estimates were imprecise, particularly in sparsely represented stages, and no individual stage pattern was interpreted as evidence of a between-stage difference. Available-case sample sizes and complete estimates are reported in Supplementary Table S7.

### 3.2. Caregiving Burden

Caregiving burden was assessed using the OCBS, which measures both the time required and the difficulty of 15 caregiving tasks on a 5-point Likert scale. In the difficulty subdomain, the ‘ventilation’ group reported significantly higher scores compared with the ‘no ventilation’ group (39.6 vs. 33.1, *p* = 0.023). In the time subdomain, scores were also higher in the ‘ventilation’ group (42.5 vs. 39.1), although this difference did not reach statistical significance (*p* = 0.073).

### 3.3. Productivity

Work Productivity and Activity Impairment (WPAI) scores did not differ significantly between ventilation groups. Mean overall work impairment was 61.3% in the ventilated group and 56.4% in the unventilated group (*p* = 0.55). Absenteeism was 11.7% and 19.2%, respectively (*p* = 0.22), and productivity loss while working was 54.3% in the ventilated group and 48.1% in the unventilated group (*p* = 0.33). None of these between-group differences was statistically significant.

### 3.4. Health-Related Quality of Life (EQ-5D-5L)

EQ-5D-5L scores described caregivers’ health-related quality of life. Mean scores were lower in the ‘ventilation’ group compared with the ‘no ventilation’ group (0.79 vs. 0.84), though this difference did not reach statistical significance (*p* = 0.056).

### 3.5. Depressive Symptoms (BDI-II)

Depressive symptom severity was assessed using the BDI-II. Among caregivers with complete BDI-II data, 46 of 54 (85.2%) in the unventilated group and 13 of 19 (68.4%) in the ventilated group had scores ≥ 14, indicating clinically relevant depressive symptoms. The odds ratio for a BDI-II score ≥ 14 in the ventilated group compared with the unventilated group was 0.38 (95% CI 0.095–1.59). The difference was not statistically significant (Fisher’s exact test, *p* = 0.17).

## 4. Discussion

This cross-sectional study found substantial caregiver burden in both ventilation groups, with higher mean OCBS difficulty scores among caregivers of ventilated patients. Clinically relevant depressive symptoms were common but were less frequent in the ventilated group. This difference was not statistically significant, its confidence interval was wide, and it should not be interpreted as a protective effect of ventilation. The greater difficulty reported by the ventilated group is consistent with multinational evidence describing substantial burden among caregivers of people with non-ambulatory and ventilation-dependent DMD [4,5].

The unexpected direction of the depression estimate may reflect sampling variation, differences in support or expectations, participation effects, or adaptation to advanced disease. In a longitudinal study of mothers caring for children with Duchenne or Becker muscular dystrophy, resilience and perceived positive impact predicted psychological adaptation, whereas child functional status and caregiver burden did not [16]. These factors were not measured in the present study, so this interpretation remains tentative.

These data describe caregivers at different functional stages at one point in time. They are broadly consistent with modelling by Landfeldt and Sandhu, which estimated greater caregiver disutility in later DMD states [17]. However, this study did not follow caregivers through stage transitions and cannot establish changes within individuals over time.

After adjustment, higher OCBS difficulty and time scores remained associated with lower EQ-5D-5L scores, whereas the association with BDI-II was attenuated and no longer statistically significant. Previous DMD research has linked caregiver anxiety and depression to perceived patient health, caregiving time and financial strain [5]. An East Asian systematic review similarly identified emotional health, family relationships, daily functioning and financial strain as central dimensions of quality of life among caregivers of children with complex health needs [18]. Further research should evaluate whether practical support, peer support and mental health interventions improve caregiver outcomes.

WPAI outcomes did not differ significantly by ventilation status. Work and activity impairment were common in both groups, suggesting that occupational effects are not determined by ventilation status alone. This is congruent with evidence from other rare neurological conditions that likewise identifies informal care and productivity loss as components of the wider economic burden [19].

No established minimal clinically important difference is available for the OCBS, so the practical importance of the observed difference is uncertain. The modest difference in caregiver EQ-5D-5L scores was also not statistically significant. Xu et al. reported convergent and known-group validity for caregiver-proxy EQ-5D-5L ratings of children and adolescents with DMD or spinal muscular atrophy, but this does not establish validity for caregivers’ own health-related quality of life [20]. Measurement limitations have also been reported in other rare neurological conditions, where existing instruments may not fully capture sleep and psychosocial burden [21]. Small samples and imprecise burden estimates are common in the DMD caregiver literature [4].

Comparable caregiver burden has been reported in other complex paediatric neurological conditions. Reviews in spinal muscular atrophy and cerebral palsy describe reduced caregiver quality of life, depression, anxiety and productivity loss [22,23]. Australian caregivers of people with developmental and epileptic encephalopathies have reported similar effects on quality of life, mental health and employment [24].

### 4.1. Implications

This study contributes Australian evidence to the international literature on DMD caregiving. The greater caregiving difficulty reported by caregivers of ventilated patients supports access to practical and psychosocial assistance for families managing respiratory support. The high prevalence of clinically relevant depressive symptoms in both groups indicates that psychological assessment and support should not be limited by ventilation status. Caregiver mental health, burden and health-related quality of life should be considered alongside patient outcomes in multidisciplinary DMD care.

### 4.2. Limitations

DMD diagnosis was caregiver-reported and was not independently verified against clinical or genetic records. Repeated IP addresses were reviewed, but an IP address is an imperfect proxy for an individual or household. Internally consistent pairs that could represent two caregivers from one household were retained to avoid systematically excluding two-caregiver families. Household identity could not be verified, so some observations may remain correlated within families.

The sample may not represent the broader Australian DMD caregiver population. Recruitment through advocacy organisations and participant networks may have favoured caregivers who were more engaged with these groups. Participation bias may also have contributed to the predominance of parents of younger children and limited comparisons across later DMD stages [15].

The regression analyses were exploratory and were constrained by small, markedly uneven stage-specific samples, including only one participant at Stage 6. Overall models adjusted for available demographic covariates, but the sample did not support more detailed adjustment for relationship to the patient or number of carers. Within-stage models remained unadjusted, individual coefficients may be unstable, and non-significant interaction tests should not be interpreted as evidence that associations are identical across stages. Multiple comparisons were not adjusted, so these estimates are hypothesis-generating.

Ventilation status was selected as a clinically meaningful marker of respiratory support needs, but DMD severity is multidimensional. Functional impairment, feeding difficulties, cardiac status and disease duration may also influence caregiver burden. The simplified caregiver-reported stages may therefore conceal important heterogeneity within both stage and ventilation groups.

Recall bias may have affected responses concerning experiences during the preceding 12 months.

All outcomes were self-reported and may have been influenced by the caregiver’s emotional state at survey completion. The survey was piloted before implementation.

## 5. Conclusions

This study found substantial psychosocial burden among Australian caregivers of people with DMD. At the time of the survey, caregivers of ventilated patients reported greater OCBS difficulty than caregivers of unventilated patients. Clinically relevant depressive symptoms were common in both groups and did not differ significantly. The clinical importance of the observed burden difference remains uncertain.

Caregiver burden, psychological symptoms and health-related quality of life should be considered across DMD care. Longitudinal studies should examine caregiver outcomes around changes in respiratory support and evaluate function, feeding, cardiac status, disease duration and caregiver and household factors with adequate representation across the clinical spectrum.

## Figures and Tables

**Table 1:** Classification of Duchenne muscular dystrophy (DMD) severity into seven stages (0–6).

Stage	Criteria for Classification
6	Patient requires full ventilation support
5	Patient requires intermittent ventilation support
4	Patient unable to feed self
3	Patient unable to remain standing for at least three seconds
2	Patient unable to walk or run without aid
1	Patient unable to get up without aid
0	None of the above apply

**Table 2:** Sociodemographic and clinical characteristics of patients with DMD.

Patient Characteristics (*n* = 73)	Mean (SD; Range)
Age, years	10.8 (6.2; 2–34)
Age at first occurrence of symptoms	Frequency (%)
<1	10 (14%)
1	10 (14%)
2	13 (18%)
3	15 (21%)
4	15 (21%)
Older than 4	10 (14%)
Patient’s ventilation status	Frequency (%)
Unventilated	54 (74%)
Ventilated	19 (26%)

## Data Availability

The datasets generated and analysed during the current study are not publicly available due to participant confidentiality but a de-identified dataset is available from the corresponding author on reasonable request.

## Supplementary Materials

The following supporting information can be downloaded at: https://www.mdpi.com/article/10.3390/healthcare14193352/s1, Table S1: EQ-5D-5L models with BDI-II score: expanded interaction and reduced additive models; Table S2: EQ-5D-5L models with OCBS difficulty score: expanded interaction and reduced additive models; Table S3: EQ-5D-5L models with OCBS time score: expanded interaction and reduced additive models; Table S4: EQ-5D-5L models with WPAI absenteeism: expanded interaction and reduced additive models; Table S5: EQ-5D-5L models with WPAI overall work impairment: expanded interaction and reduced additive models; Table S6: EQ-5D-5L models with WPAI presenteeism: expanded interaction and reduced additive models; Table S7: Stage-specific univariate associations between caregiver-reported outcome measures and EQ-5D-5L; Table S8: OLS regression with EQ-5D score as dependent variable, caregiver-reported outcome measure as independent variable with covariates; Table S9: OLS regression with EQ-5D score as dependent variable, caregiver-reported outcome measure as independent variable with covariates; Table S10: OLS regression with EQ-5D score as dependent variable, caregiver-reported outcome measure as independent variable with covariates; Table S11: OLS regression with EQ-5D score as dependent variable, caregiver-reported outcome measure as independent variable with covariates; Table S12: OLS regression with EQ-5D score as dependent variable, caregiver-reported outcome measure as independent variable with covariates; Table S13: OLS regression with EQ-5D score as dependent variable, caregiver-reported outcome measure as independent variable with covariates; Table S14: Review of responses sharing an IP address; Table S15: Checklist for Reporting Results of Internet E-Surveys (CHERRIES); Table S16: STROBE Statement—Checklist of items that should be included in reports of cross-sectional studies.
