# Supplementary material for: Psychosocial Burden of Duchenne Muscular Dystrophy on Caregivers in Australia: A Cross-Sectional Study
Source: Healthcare (Basel). 2026 Oct 8;14(19):3352. doi: 10.3390/healthcare14193352 (PMC13665195; doi:10.3390/healthcare14193352)
Supplement: Supplementary file 1 [file healthcare-14-03352-s001.zip › healthcare-4486487-supplementary.pdf]

Supplementary Materials

Table S1. EQ-5D-5L models with BDI-II score: expanded interaction and reduced additive models.

	With interaction (N = 73)			Without interaction (N = 73)		
**Characteristic**	**Beta**	**95% CI**	**p-value**	**Beta**	**95% CI**	**p-value**
bdi_score	0.00	-0.01, 0.00	0.093	0.00	-0.01, 0.00	<0.001
dmd_stage						
0	—	—		—	—	
1	-0.08	-0.86, 0.70	0.8	0.03	-0.10, 0.16	0.6
2	-0.11	-0.30, 0.08	0.3	-0.03	-0.10, 0.05	0.5
3	0.01	-0.22, 0.25	>0.9	0.02	-0.06, 0.10	0.6
4	0.11	-0.15, 0.36	0.4	0.06	-0.04, 0.15	0.2
5	-0.01	-0.17, 0.15	0.9	-0.07	-0.14, 0.00	0.036
6	-0.12	-0.34, 0.09	0.3	-0.13	-0.34, 0.09	0.2
bdi_score * dmd_stage						
bdi_score * 1	0.01	-0.04, 0.06	0.8			
bdi_score * 2	0.00	0.00, 0.01	0.3			
bdi_score * 3	0.00	-0.01, 0.01	>0.9			
bdi_score * 4	0.00	-0.02, 0.01	0.7			
bdi_score * 5	0.00	-0.01, 0.00	0.4			
bdi_score * 6						
R^2	0.324			0.281		
Adjusted R^2	0.189			0.203		

Abbreviation: CI = Confidence Interval

$F(5,60) = 0.77$ $p = 0.57$

Table S2. EQ-5D-5L models with OCBS difficulty score: expanded interaction and reduced additive models.

	With interaction (N = 73)			Without interaction (N = 73)		
**Characteristic**	**Beta**	**95% CI**	**p-value**	**Beta**	**95% CI**	**p-value**
ocbs_difficulty_score	0.00	-0.01, 0.00	0.087	0.00	-0.01, 0.00	<0.001
dmd_stage						
0	—	—		—	—	
1	0.04	-0.75, 0.84	>0.9	0.04	-0.09, 0.17	0.6
2	-0.22	-0.51, 0.06	0.12	-0.02	-0.09, 0.06	0.7
3	0.13	-0.26, 0.52	0.5	0.00	-0.08, 0.08	>0.9
4	0.26	-0.24, 0.76	0.3	0.12	0.02, 0.22	0.017
5	0.13	-0.13, 0.38	0.3	-0.02	-0.09, 0.05	0.6
6	-0.10	-0.31, 0.11	0.3	-0.10	-0.31, 0.11	0.4
ocbs_difficulty_score * dmd_stage						
ocbs_difficulty_score * 1	0.00	-0.03, 0.03	>0.9			
ocbs_difficulty_score * 2	0.01	0.00, 0.01	0.14			
ocbs_difficulty_score * 3	0.00	-0.02, 0.01	0.5			
ocbs_difficulty_score * 4	0.00	-0.02, 0.01	0.6			
ocbs_difficulty_score * 5	0.00	-0.01, 0.00	0.3			
ocbs_difficulty_score * 6						
R^2	0.343			0.268		
Adjusted R^2	0.211			0.189		

Abbreviation: CI = Confidence Interval

$F(5,60) = 1.36$ $p = 0.25$

Table S3. EQ-5D-5L models with OCBS time score: expanded interaction and reduced additive models.

Characteristic	With interaction (N = 73)			Without interaction (N = 73)		
Characteristic	Beta	95% CI	p-value	Beta	95% CI	p-value
ocbs_time_score	0.00	-0.01, 0.00	0.029	0.00	-0.01, 0.00	0.052
dmd_stage						
0	—	—		—	—	
1	-0.32	-1.9, 1.3	0.7	0.06	-0.08, 0.20	0.4
2	-0.26	-0.56, 0.03	0.076	-0.03	-0.11, 0.04	0.4
3	-0.13	-0.51, 0.24	0.5	0.02	-0.06, 0.10	0.6
4	-0.01	-0.38, 0.36	>0.9	0.09	-0.02, 0.19	0.094
5	0.06	-0.43, 0.55	0.8	-0.04	-0.12, 0.03	0.2
6	-0.11	-0.33, 0.12	0.4	-0.11	-0.34, 0.12	0.3
ocbs_time_score * dmd_stage						
ocbs_time_score * 1	0.01	-0.03, 0.05	0.6			
ocbs_time_score * 2	0.01	0.00, 0.01	0.11			
ocbs_time_score * 3	0.00	-0.01, 0.01	0.4			
ocbs_time_score * 4	0.00	-0.01, 0.01	0.6			
ocbs_time_score * 5	0.00	-0.01, 0.01	0.7			
ocbs_time_score * 6						
R^2	0.231			0.183		
Adjusted R^2	0.077			0.095		

Abbreviation: CI = Confidence Interval

$F(5,60) = 0.74$ $p = 0.59$

Table S4. EQ-5D-5L models with WPAI absenteeism: expanded interaction and reduced additive models.

	With interaction (N = 38)			Without interaction (N = 38)		
**Characteristic**	**Beta**	**95% CI**	**p-value**	**Beta**	**95% CI**	**p-value**
WPAI_absenteeism	0.13	-0.08, 0.34	0.2	0.08	-0.09, 0.26	0.3
dmd_stage						
0	—	—		—	—	
1	0.08	-0.09, 0.26	0.3	0.07	-0.07, 0.21	0.3
2	0.01	-0.16, 0.18	>0.9	-0.01	-0.13, 0.11	0.9
3	0.02	-0.15, 0.18	0.8	0.03	-0.08, 0.14	0.6
4	0.12	-0.06, 0.30	0.2	0.09	-0.05, 0.23	0.2
5	-0.04	-0.17, 0.09	0.5	-0.10	-0.20, -0.01	0.033
6	-0.14	-0.36, 0.09	0.2	-0.14	-0.35, 0.08	0.2
WPAI_absenteeism * dmd_stage						
WPAI_absenteeism * 1	0.05	-1.7, 1.8	>0.9			
WPAI_absenteeism * 2	-0.08	-0.57, 0.41	0.7			
WPAI_absenteeism * 3	0.23	-0.86, 1.3	0.7			
WPAI_absenteeism * 4	-0.41	-2.0, 1.2	0.6			
WPAI_absenteeism * 5	-0.54	-1.3, 0.19	0.14			
WPAI_absenteeism * 6						
R^2	0.426			0.360		
Adjusted R^2	0.150			0.210		

Abbreviation: CI = Confidence Interval

$F(5,25) = 0.57$ $p = 0.72$

Table S5. EQ-5D-5L models with WPAI overall work impairment: expanded interaction and reduced additive models.

	With interaction (N = 38)			Without interaction (N = 38)		
**Characteristic**	**Beta**	**95% CI**	**p-value**	**Beta**	**95% CI**	**p-value**
WPAI_overall_work_impairment	0.05	-0.13, 0.23	0.6	0.04	-0.10, 0.17	0.6
dmd_stage						
0	—	—		—	—	
1	0.04	-0.59, 0.68	0.9	0.05	-0.08, 0.19	0.4
2	-0.09	-0.57, 0.39	0.7	-0.02	-0.14, 0.10	0.7
3	-0.01	-0.26, 0.24	>0.9	0.02	-0.09, 0.13	0.8
4	0.06	-0.37, 0.48	0.8	0.07	-0.06, 0.21	0.3
5	-0.02	-0.28, 0.25	0.9	-0.12	-0.21, -0.03	0.010
6	-0.14	-0.38, 0.09	0.2	-0.14	-0.36, 0.08	0.2
WPAI_overall_work_impairment * dmd_stage						
WPAI_overall_work_impairment * 1	0.01	-0.95, 0.97	>0.9			
WPAI_overall_work_impairment * 2	0.10	-0.61, 0.81	0.8			
WPAI_overall_work_impairment * 3	0.05	-0.37, 0.48	0.8			
WPAI_overall_work_impairment * 4	0.06	-1.0, 1.1	>0.9			
WPAI_overall_work_impairment * 5	-0.18	-0.60, 0.25	0.4			
WPAI_overall_work_impairment * 6						
R^2	0.372			0.345		
Adjusted R^2	0.071			0.193		

Abbreviation: CI = Confidence Interval

$F(5,25) = 0.21$ $p = 0.95$

Table S6. EQ-5D-5L models with WPAI presenteeism: expanded interaction and reduced additive models.

	With interaction (N = 51)			Without interaction (N = 51)		
**Characteristic**	**Beta**	**95% CI**	**p-value**	**Beta**	**95% CI**	**p-value**
WPAI_presenteeism	0.05	-0.15, 0.25	0.6	0.04	-0.10, 0.17	0.6
dmd_stage						
0	—	—		—	—	
1	0.04	-0.65, 0.73	>0.9	0.05	-0.09, 0.18	0.5
2	-0.09	-0.30, 0.12	0.4	-0.06	-0.15, 0.04	0.2
3	0.00	-0.24, 0.24	>0.9	0.01	-0.09, 0.11	0.9
4	-0.01	-0.50, 0.48	>0.9	0.07	-0.07, 0.21	0.3
5	0.03	-0.21, 0.26	0.8	-0.09	-0.17, -0.01	0.026
6	-0.15	-0.38, 0.09	0.2	-0.14	-0.37, 0.08	0.2
WPAI_presenteeism * dmd_stage						
WPAI_presenteeism * 1	0.01	-1.1, 1.1	>0.9			
WPAI_presenteeism * 2	0.08	-0.34, 0.49	0.7			
WPAI_presenteeism * 3	0.02	-0.39, 0.42	>0.9			
WPAI_presenteeism * 4	0.25	-1.2, 1.7	0.7			

	With interaction (N = 51)			Without interaction (N = 51)		
**Characteristic**	**Beta**	**95% CI**	**p-value**	**Beta**	**95% CI**	**p-value**
WPAI_presenteeism * 5	-0.23	-0.65, 0.20	0.3			
WPAI_presenteeism * 6						
R^2	0.253			0.218		
Adjusted R^2	0.017			0.090		

Abbreviation: CI = Confidence Interval

$F(5,38) = 0.36$ $p = 0.87$

Table S7. Stage-specific univariate associations between caregiver-reported outcome measures and EQ-5D-5L.

Characteristic	Beta	95% CI	p-value
OCBS Difficulty Score — DMD Stage 0			
ocbs_difficulty_score	-0.0042	-0.0090, 0.0006	0.084
No. Obs.	22		
R^2	0.142		
OCBS Difficulty Score — DMD Stage 1			
ocbs_difficulty_score	-0.0042	-0.0279, 0.0196	0.3
No. Obs.	3		
R^2	0.834		
OCBS Difficulty Score — DMD Stage 2			
ocbs_difficulty_score	0.0018	-0.0082, 0.0118	0.7
No. Obs.	13		
R^2	0.014		
OCBS Difficulty Score — DMD Stage 3			
ocbs_difficulty_score	-0.0085	-0.0160, -0.0010	0.030
No. Obs.	10		
R^2	0.463		
OCBS Difficulty Score — DMD Stage 4			
ocbs_difficulty_score	-0.0079	-0.0159, 0.0001	0.052
No. Obs.	6		
R^2	0.652		
OCBS Difficulty Score — DMD Stage 5			
ocbs_difficulty_score	-0.0079	-0.0129, -0.0030	0.004
No. Obs.	18		
R^2	0.420		
OCBS Difficulty Score — DMD Stage 6			
ocbs_difficulty_score			
No. Obs.	1		
R^2	0.000		
OCBS Time Score — DMD Stage 0			
ocbs_time_score	-0.0049	-0.0086, -0.0012	0.012
No. Obs.	22		
R^2	0.274		
OCBS Time Score — DMD Stage 1			
ocbs_time_score	0.0055	-0.0369, 0.0478	0.3
No. Obs.	3		
R^2	0.729		
OCBS Time Score — DMD Stage 2			
ocbs_time_score	0.0012	-0.0075, 0.0099	0.8

Characteristic	Beta	95% CI	p-value
No. Obs.	13		
R^2	0.008		
OCBS Time Score — DMD Stage 3			
ocbs_time_score	-0.0012	-0.0074, 0.0051	0.7
No. Obs.	10		
R^2	0.022		
OCBS Time Score — DMD Stage 4			
ocbs_time_score	-0.0025	-0.0102, 0.0052	0.4
No. Obs.	6		
R^2	0.170		
OCBS Time Score — DMD Stage 5			
ocbs_time_score	-0.0073	-0.0205, 0.0060	0.3
No. Obs.	18		
R^2	0.078		
OCBS Time Score — DMD Stage 6			
ocbs_time_score			
No. Obs.	1		
R^2	0.000		
BDI Score — DMD Stage 0			
bdi_score	-0.0043	-0.0093, 0.0007	0.086
No. Obs.	22		
R^2	0.141		
BDI Score — DMD Stage 1			
bdi_score	0.0030	-0.0850, 0.0910	0.7
No. Obs.	3		
R^2	0.158		
BDI Score — DMD Stage 2			
bdi_score	-0.0007	-0.0093, 0.0079	0.9
No. Obs.	13		
R^2	0.003		
BDI Score — DMD Stage 3			
bdi_score	-0.0040	-0.0099, 0.0020	0.2
No. Obs.	10		
R^2	0.229		
BDI Score — DMD Stage 4			
bdi_score	-0.0071	-0.0168, 0.0025	0.11
No. Obs.	6		
R^2	0.513		

Characteristic	Beta	95% CI	p-value
BDI Score — DMD Stage 5			
bdi_score	-0.0073	-0.0118, -0.0028	0.003
No. Obs.	18		
R^2	0.423		
BDI Score — DMD Stage 6			
bdi_score			
No. Obs.	1		
R^2	0.000		
WPAI Overall Work Impairment — DMD Stage 0			
WPAI_overall_work_impairment	0.0488	-0.1321, 0.2298	0.6
No. Obs.	12		
R^2	0.035		
WPAI Overall Work Impairment — DMD Stage 1			
WPAI_overall_work_impairment	0.0583	-1.5071, 1.6237	0.7
No. Obs.	3		
R^2	0.183		
WPAI Overall Work Impairment — DMD Stage 2			
WPAI_overall_work_impairment	0.1523	-1.5873, 1.8919	0.7
No. Obs.	4		
R^2	0.066		
WPAI Overall Work Impairment — DMD Stage 3			
WPAI_overall_work_impairment	0.1021	-0.3045, 0.5087	0.5
No. Obs.	5		
R^2	0.175		
WPAI Overall Work Impairment — DMD Stage 4			
WPAI_overall_work_impairment	0.1043	-4.8003, 5.0089	0.8
No. Obs.	3		
R^2	0.068		
WPAI Overall Work Impairment — DMD Stage 5			
WPAI_overall_work_impairment	-0.1262	-0.6374, 0.3851	0.6
No. Obs.	10		
R^2	0.039		
WPAI Overall Work Impairment — DMD Stage 6			
WPAI_overall_work_impairment			
No. Obs.	1		
R^2	0.000		
WPAI Absenteeism — DMD Stage 0			
WPAI_absenteeism	0.1315	-0.0715, 0.3345	0.2

Characteristic	Beta	95% CI	p-value
No. Obs.	12		
R^2	0.172		
WPAI Absenteeism — DMD Stage 1			
WPAI_absenteeism	0.1800	-2.3142, 2.6742	0.5
No. Obs.	3		
R^2	0.457		
WPAI Absenteeism — DMD Stage 2			
WPAI_absenteeism	0.0504	-1.1426, 1.2434	0.9
No. Obs.	4		
R^2	0.016		
WPAI Absenteeism — DMD Stage 3			
WPAI_absenteeism	0.3628	-0.7712, 1.4968	0.4
No. Obs.	5		
R^2	0.257		
WPAI Absenteeism — DMD Stage 4			
WPAI_absenteeism	-0.2760	-7.5386, 6.9866	0.7
No. Obs.	3		
R^2	0.189		
WPAI Absenteeism — DMD Stage 5			
WPAI_absenteeism	-0.4087	-1.3585, 0.5410	0.4
No. Obs.	10		
R^2	0.110		
WPAI Absenteeism — DMD Stage 6			
WPAI_absenteeism			
No. Obs.	1		
R^2	0.000		
WPAI Presenteeism — DMD Stage 0			
WPAI_presenteeism	0.0512	-0.1255, 0.2279	0.5
No. Obs.	17		
R^2	0.025		
WPAI Presenteeism — DMD Stage 1			
WPAI_presenteeism	0.0600	-1.7006, 1.8206	0.7
No. Obs.	3		
R^2	0.158		
WPAI Presenteeism — DMD Stage 2			
WPAI_presenteeism	0.1269	-0.4723, 0.7261	0.6
No. Obs.	8		
R^2	0.043		

**Characteristic**	**Beta**	**95% CI**	**p-value**
WPAI Presenteeism — DMD Stage 3			
WPAI_presenteeism	0.0682	-0.2281, 0.3646	0.6
No. Obs.	6		
R^2	0.093		
WPAI Presenteeism — DMD Stage 4			
WPAI_presenteeism	0.2975	-5.2595, 5.8545	0.6
No. Obs.	3		
R^2	0.316		
WPAI Presenteeism — DMD Stage 5			
WPAI_presenteeism	-0.1771	-0.6406, 0.2864	0.4
No. Obs.	13		
R^2	0.060		
WPAI Presenteeism — DMD Stage 6			
WPAI_presenteeism			
No. Obs.	1		
R^2	0.000		

Abbreviation: CI = Confidence Interval

Table S8. OLS regression with EQ-5D score as dependent variable, caregiver-reported outcome measure as independent variable with covariates.

**Characteristic**	**Beta**	**95% CI**	**p-value**
ocbs_difficulty_score	-0.0048	-0.0075, -0.0021	<0.001
dmd_stage			
0	—	—	
1	0.0755	-0.0490, 0.2000	0.2
2	-0.0329	-0.1036, 0.0378	0.4
3	-0.0280	-0.1132, 0.0571	0.5
4	0.0504	-0.0479, 0.1487	0.3
5	-0.0711	-0.1470, 0.0048	0.066
6	-0.2154	-0.4407, 0.0099	0.061
carer_age			
18 to 24 years old	—	—	
25 to 34 years old	0.0214	-0.1401, 0.1828	0.8
35 to 44 years old	-0.0389	-0.1890, 0.1112	0.6
45 to 55 years old	0.0511	-0.1059, 0.2082	0.5
55 to 64 years old	-0.0157	-0.1848, 0.1534	0.9
gender			
Female	—	—	
Male	0.0861	0.0316, 0.1405	0.002
age	0.0039	-0.0020, 0.0098	0.2
employed			
No	—	—	
Yes	-0.0092	-0.0666, 0.0483	0.8
No. Obs.	73		
R^2	0.475		

Abbreviation: CI = Confidence Interval

Table S9. OLS regression with EQ-5D score as dependent variable, caregiver-reported outcome measure as independent variable with covariates.

**Characteristic**	**Beta**	**95% CI**	**p-value**
ocbs_time_score	-0.0030	-0.0058, -0.0002	0.036
dmd_stage			
0	—	—	
1	0.1010	-0.0296, 0.2316	0.13
2	-0.0474	-0.1243, 0.0294	0.2
3	-0.0151	-0.1049, 0.0747	0.7
4	0.0173	-0.0863, 0.1210	0.7
5	-0.0983	-0.1782, -0.0184	0.017
6	-0.2417	-0.4812, -0.0021	0.048
carer_age			
18 to 24 years old	—	—	
25 to 34 years old	0.0725	-0.0947, 0.2397	0.4
35 to 44 years old	-0.0065	-0.1646, 0.1516	>0.9
45 to 55 years old	0.0941	-0.0719, 0.2601	0.3
55 to 64 years old	0.0136	-0.1653, 0.1924	0.9
gender			
Female	—	—	
Male	0.0750	0.0172, 0.1329	0.012
age	0.0047	-0.0016, 0.0110	0.14
employed			
No	—	—	
Yes	-0.0312	-0.0942, 0.0318	0.3
No. Obs.	73		
R^2	0.409		

Abbreviation: CI = Confidence Interval

Table S10. OLS regression with EQ-5D score as dependent variable, caregiver-reported outcome measure as independent variable with covariates.

**Characteristic**	**Beta**	**95% CI**	**p-value**
bdi_score	-0.0025	-0.0057, 0.0007	0.12
dmd_stage			
0	—	—	
1	0.0940	-0.0414, 0.2295	0.2
2	-0.0284	-0.1047, 0.0480	0.5
3	0.0127	-0.0798, 0.1052	0.8
4	0.0224	-0.0829, 0.1277	0.7
5	-0.0836	-0.1652, -0.0020	0.045
6	-0.1905	-0.4377, 0.0568	0.13
carer_age			
18 to 24 years old	—	—	
25 to 34 years old	0.0690	-0.1023, 0.2403	0.4
35 to 44 years old	-0.0136	-0.1748, 0.1476	0.9
45 to 55 years old	0.0510	-0.1221, 0.2242	0.6
55 to 64 years old	-0.0140	-0.1987, 0.1706	0.9
gender			
Female	—	—	
Male	0.0611	-0.0021, 0.1243	0.058
age	0.0030	-0.0036, 0.0096	0.4
employed			
No	—	—	
Yes	-0.0196	-0.0820, 0.0428	0.5
No. Obs.	73		
R^2	0.388		

Abbreviation: CI = Confidence Interval

Table S11. OLS regression with EQ-5D score as dependent variable, caregiver-reported outcome measure as independent variable with covariates.

Characteristic	Beta	95% CI	p-value
WPAI_overall_work_impairment	-0.0339	-0.1938, 0.1260	0.7
carer_age			
18 to 24 years old	—	—	
25 to 34 years old	0.1030	-0.1081, 0.3141	0.3
35 to 44 years old	-0.0178	-0.2067, 0.1710	0.8
45 to 55 years old	-0.0221	-0.2208, 0.1766	0.8
55 to 64 years old	-0.0731	-0.2915, 0.1453	0.5
gender			
Female	—	—	
Male	0.0497	-0.0331, 0.1326	0.2
age	0.0027	-0.0062, 0.0115	0.5
No. Obs.	38		
R^2	0.197		

Abbreviation: CI = Confidence Interval

Table S12. OLS regression with EQ-5D score as dependent variable, caregiver-reported outcome measure as independent variable with covariates.

**Characteristic**	**Beta**	**95% CI**	**p-value**
WPAI_absenteeism	-0.0147	-0.2186, 0.1891	0.9
carer_age			
18 to 24 years old	—	—	
25 to 34 years old	0.0957	-0.1244, 0.3157	0.4
35 to 44 years old	-0.0271	-0.2108, 0.1566	0.8
45 to 55 years old	-0.0336	-0.2243, 0.1571	0.7
55 to 64 years old	-0.0728	-0.2925, 0.1469	0.5
gender			
Female	—	—	
Male	0.0480	-0.0347, 0.1308	0.2
age	0.0026	-0.0063, 0.0115	0.6
No. Obs.	38		
R^2	0.193		

Abbreviation: CI = Confidence Interval

Table S13. OLS regression with EQ-5D score as dependent variable, caregiver-reported outcome measure as independent variable with covariates.

Characteristic	Beta	95% CI	p-value
WPAI_presenteeism	0.0191	-0.1257, 0.1639	0.8
carer_age			
18 to 24 years old	—	—	
25 to 34 years old	0.0898	-0.0966, 0.2762	0.3
35 to 44 years old	-0.0256	-0.1957, 0.1445	0.8
45 to 55 years old	-0.0127	-0.1909, 0.1656	0.9
55 to 64 years old	-0.0587	-0.2539, 0.1365	0.5
gender			
Female	—	—	
Male	0.0645	-0.0007, 0.1296	0.052
age	0.0027	-0.0050, 0.0104	0.5
No. Obs.	51		
R^2	0.201		

Abbreviation: CI = Confidence Interval

Table S14. Review of responses sharing an IP address.

Masked IP address	Responses	Assessment	Analytical decision	Records excluded
103.xxx.xxx.xxx	2	Plausible separate caregivers in one household	Retained both responses	0
116.xxx.xxx.xxx	2	Contradictory repeated responses	Excluded both responses	2
120.xxx.xxx.xxx	2	Incomplete response matched a completed response	Excluded the incomplete response	1
14.xxx.xxx.xxx (A)	2	Plausible separate caregivers in one household	Retained both responses	0
14.xxx.xxx.xxx (B)	2	Contradictory repeated responses	Excluded both responses	2
144.xxx.xxx.xxx	2	Contradictory repeated responses	Excluded both responses	2
210.xxx.xxx.xxx	3	Contradictory repeated responses	Excluded all three responses	3
61.xxx.xxx.xxx	2	Plausible separate caregivers in one household	Retained both responses	0

Notes: Repeated IP addresses were treated as signals for review rather than proof of duplicate participation. Internally consistent pairs that could represent two caregivers in one household were retained. A total of 10 records were excluded before reanalysis.

Table S15. Checklist for Reporting Results of Internet E-Surveys (CHERRIES).

Item Category	Checklist Item	Explanation	Page/section
**Design**			
	Describe survey design	Describe target population, sample frame. Is the sample a convenience sample? (In "open" surveys this is most likely.)	pp. 2–3, Section 2.1
**IRB (Institutional Review Board) approval and informed consent process**			
	IRB approval	Mention whether the study has been approved by an IRB.	p. 2, Section 2.1; p. 10, Institutional Review Board Statement
	Informed consent	Describe the informed consent process. Where were the participants told the length of time of the survey, which data were stored and where and for how long, who the investigator was, and the purpose of the study?	p. 2, Section 2.1; p. 10, Informed Consent Statement
	Data protection	If any personal information was collected or stored, describe what mechanisms were used to protect unauthorized access.	Not reported (data-security procedures)
**Development and pre-testing**			
	Development and testing	State how the survey was developed, including whether the usability and technical functionality of the electronic questionnaire had been tested before fielding the questionnaire.	p. 9, Section 4.2, final paragraph
**Recruitment process and description of the sample having access to the questionnaire**			
	Open survey versus closed survey	An "open survey" is a survey open for each visitor of a site, while a closed survey is only open to a sample which the investigator knows (password-protected survey).	Not explicitly reported
	Contact mode	Indicate whether or not the initial contact with the potential participants was made on the Internet. (Investigators may also send out questionnaires by mail and allow for Web-based data entry.)	p. 2, Section 2.1
	Advertising the survey	How/where was the survey announced or advertised? Some examples are offline media (newspapers), or online (mailing lists – If yes, which ones?) or banner ads (Where were these banner ads posted and what did they look like?). It is important to know the wording of the announcement as it will heavily influence who chooses to participate. Ideally the survey announcement should be published as an appendix.	p. 2, Section 2.1; p. 10, Acknowledgments
**Survey administration**			

Item Category	Checklist Item	Explanation	Page/section
	Web/E-mail	State the type of e-survey (eg, one posted on a Web site, or one sent out through e-mail). If it is an e-mail survey, were the responses entered manually into a database, or was there an automatic method for capturing responses?	p. 2, Section 2.1 (Qualtrics survey)
	Context	Describe the Web site (for mailing list/newsgroup) in which the survey was posted. What is the Web site about, who is visiting it, what are visitors normally looking for? Discuss to what degree the content of the Web site could pre-select the sample or influence the results. For example, a survey about vaccination on a anti-immunization Web site will have different results from a Web survey conducted on a government Web site	p. 2, Section 2.1; p. 9, Section 4.2 (recruitment and participation bias)
	Mandatory/voluntary	Was it a mandatory survey to be filled in by every visitor who wanted to enter the Web site, or was it a voluntary survey?	p. 2, Section 2.1 (informed consent and recruitment)
	Incentives	Were any incentives offered (eg, monetary, prizes, or non-monetary incentives such as an offer to provide the survey results)?	p. 2, Section 2.1 (compensation)
	Time/Date	In what timeframe were the data collected?	p. 2, Section 2.1 (survey dates)
	Randomization of items or questionnaires	To prevent biases items can be randomized or alternated.	Not reported
	Adaptive questioning	Use adaptive questioning (certain items, or only conditionally displayed based on responses to other items) to reduce number and complexity of the questions.	Not reported
	Number of Items	What was the number of questionnaire items per page? The number of items is an important factor for the completion rate.	Not reported
	Number of screens (pages)	Over how many pages was the questionnaire distributed? The number of items is an important factor for the completion rate.	Not reported
	Completeness check	It is technically possible to do consistency or completeness checks before the questionnaire is submitted. Was this done, and if “yes”, how (usually JAVAScript)? An alternative is to check for completeness after the questionnaire has been submitted (and highlight mandatory items). If this has been done, it should be reported. All items should provide a non-response option such as “not applicable” or “rather not say”, and selection of one response option should be enforced.	Not reported
	Review step	State whether respondents were able to review and change their answers (eg, through a Back button or a Review step which displays a summary of the responses and asks the respondents if they are correct).	Not reported

Item Category	Checklist Item	Explanation	Page/section
		**Response rates**	
	Unique site visitor	If you provide view rates or participation rates, you need to define how you determined a unique visitor. There are different techniques available, based on IP addresses or cookies or both.	Not available
	View rate (Ratio of unique survey visitors/unique site visitors)	Requires counting unique visitors to the first page of the survey, divided by the number of unique site visitors (not page views!). It is not unusual to have view rates of less than 0.1 % if the survey is voluntary.	Not available
	Participation rate (Ratio of unique visitors who agreed to participate/unique first survey page visitors)	Count the unique number of people who filled in the first survey page (or agreed to participate, for example by checking a checkbox), divided by visitors who visit the first page of the survey (or the informed consents page, if present). This can also be called “recruitment” rate.	Not available
	Completion rate (Ratio of users who finished the survey/users who agreed to participate)	The number of people submitting the last questionnaire page, divided by the number of people who agreed to participate (or submitted the first survey page). This is only relevant if there is a separate “informed consent” page or if the survey goes over several pages. This is a measure for attrition. Note that “completion” can involve leaving questionnaire items blank. This is not a measure for how completely questionnaires were filled in. (If you need a measure for this, use the word “completeness rate”.)	Not available
		**Preventing multiple entries from the same individual**	
	Cookies used	Indicate whether cookies were used to assign a unique user identifier to each client computer. If so, mention the page on which the cookie was set and read, and how long the cookie was valid. Were duplicate entries avoided by preventing users access to the survey twice; or were duplicate database entries having the same user ID eliminated before analysis? In the latter case, which entries were kept for analysis (eg, the first entry or the most recent)?	Not reported
	IP check	Indicate whether the IP address of the client computer was used to identify potential duplicate entries from the same user. If so, mention the period of time for which no two entries from the same IP address were allowed (eg, 24 hours). Were duplicate entries avoided by preventing users with the same IP address access to the survey twice; or were duplicate database entries having the same IP address within a given period of time eliminated before analysis? If the latter, which entries were kept for analysis (eg, the first entry or the most recent)?	p. 3, Section 2.1 (IP-address review); Supplementary Table S14

Item Category	Checklist Item	Explanation	Page/section
	Log file analysis	Indicate whether other techniques to analyze the log file for identification of multiple entries were used. If so, please describe.	Not reported
	Registration	In "closed" (non-open) surveys, users need to login first and it is easier to prevent duplicate entries from the same user. Describe how this was done. For example, was the survey never displayed a second time once the user had filled it in, or was the username stored together with the survey results and later eliminated? If the latter, which entries were kept for analysis (eg, the first entry or the most recent)?	Not reported
		**Analysis**	
	Handling of incomplete questionnaires	Were only completed questionnaires analyzed? Were questionnaires which terminated early (where, for example, users did not go through all questionnaire pages) also analyzed?	p. 3, Section 2.1; p. 4, Section 2.4; Supplementary Tables S1–S14
	Questionnaires submitted with an atypical timestamp	Some investigators may measure the time people needed to fill in a questionnaire and exclude questionnaires that were submitted too soon. Specify the timeframe that was used as a cut-off point, and describe how this point was determined.	Not reported
	Statistical correction	Indicate whether any methods such as weighting of items or propensity scores have been used to adjust for the non-representative sample; if so, please describe the methods.	Not reported (sampling weights or other representativeness correction)

Table S16. STROBE Statement - Checklist of items that should be included in reports of cross-sectional studies

	Item No	Recommendation	Page/section
**Title and abstract**	1	(*a*) Indicate the study's design with a commonly used term in the title or the abstract	p. 1, title
		(*b*) Provide in the abstract an informative and balanced summary of what was done and what was found	p. 1, Abstract
**Introduction**			
Background/rationale	2	Explain the scientific background and rationale for the investigation being reported	pp. 1–2, Section 1
Objectives	3	State specific objectives, including any prespecified hypotheses	p. 2, Section 1, final paragraph
**Methods**			
Study design	4	Present key elements of study design early in the paper	pp. 2–3, Section 2.1
Setting	5	Describe the setting, locations, and relevant dates, including periods of recruitment, exposure, follow-up, and data collection	pp. 2–3, Section 2.1
Participants	6	(*a*) Give the eligibility criteria, and the sources and methods of selection of participants	pp. 2–3, Section 2.1
Variables	7	Clearly define all outcomes, exposures, predictors, potential confounders, and effect modifiers. Give diagnostic criteria, if applicable	pp. 3–4, Sections 2.2–2.4; Table 1
Data sources/ measurement	8*	For each variable of interest, give sources of data and details of methods of assessment (measurement). Describe comparability of assessment methods if there is more than one group	pp. 3–4, Sections 2.2–2.3; Table 1
Bias	9	Describe any efforts to address potential sources of bias	p. 3, Section 2.1; p. 9, Section 4.2; Supplementary Table S14
Study size	10	Explain how the study size was arrived at	p. 3, Section 2.1, final paragraph
Quantitative variables	11	Explain how quantitative variables were handled in the analyses. If applicable, describe which groupings were chosen and why	p. 4, Sections 2.2.4–2.4; Table 1; p. 5, Table 3
Statistical methods	12	(*a*) Describe all statistical methods, including those used to control for confounding	p. 4, Section 2.4
		(*b*) Describe any methods used to examine subgroups and interactions	p. 4, Section 2.4 (within-stage models and interaction tests)
		(*c*) Explain how missing data were addressed	p. 4, Section 2.4 (available-case analysis); p. 6, Section 3.1; Supplementary Tables S1–S13
		(*d*) If applicable, describe analytical methods taking account of sampling strategy	Not reported
		(*e*) Describe any sensitivity analyses	Not reported

	Item No	Recommendation	Page/section
**Results**			
Participants	13*	(a) Report numbers of individuals at each stage of study—eg numbers potentially eligible, examined for eligibility, confirmed eligible, included in the study, completing follow-up, and analysed	p. 3, Section 2.1; pp. 4–5, Section 3.1 and Tables 2–3; Supplementary Tables S1–S14
		(b) Give reasons for non-participation at each stage	Not available
		(c) Consider use of a flow diagram	Not included
Descriptive data	14*	(a) Give characteristics of study participants (eg demographic, clinical, social) and information on exposures and potential confounders	pp. 4–5, Section 3.1 and Tables 2–3; p. 6, Figure 1
		(b) Indicate number of participants with missing data for each variable of interest	Partly reported: p. 6, Section 3.1; Supplementary Tables S1–S13 (analytic n)
Outcome data	15*	Report numbers of outcome events or summary measures	pp. 6–8, Sections 3.1–3.5
Main results	16	(*a*) Give unadjusted estimates and, if applicable, confounder-adjusted estimates and their precision (eg, 95% confidence interval). Make clear which confounders were adjusted for and why they were included	p. 4, Section 2.4; pp. 6–8, Sections 3.1–3.5; Supplementary Tables S1–S13
		(*b*) Report category boundaries when continuous variables were categorized	p. 4, Sections 2.2.4–2.3 and Table 1; p. 5, Table 3
		(*c*) If relevant, consider translating estimates of relative risk into absolute risk for a meaningful time period	pp. 7–8, Section 3.5 (group proportions)
Other analyses	17	Report other analyses done—eg analyses of subgroups and interactions, and sensitivity analyses	pp. 6–7, Section 3.1 and Figures 2–3; Supplementary Tables S1–S13
**Discussion**			
Key results	18	Summarise key results with reference to study objectives	p. 8, Section 4
Limitations	19	Discuss limitations of the study, taking into account sources of potential bias or imprecision. Discuss both direction and magnitude of any potential bias	p. 9, Section 4.2
Interpretation	20	Give a cautious overall interpretation of results considering objectives, limitations, multiplicity of analyses, results from similar studies, and other relevant evidence	pp. 8–9, Sections 4–4.2
Generalisability	21	Discuss the generalisability (external validity) of the study results	p. 9, Section 4.2, second paragraph
**Other information**			
Funding	22	Give the source of funding and the role of the funders for the present study and, if applicable, for the original study on which the present article is based	p. 10, Funding and Conflicts of Interest
